# Long-Term Interannual and Seasonal Links between the Nutrient Regime, Sestonic Chlorophyll and Dominant Bluegreen Algae under the Varying Intensity of Monsoon Precipitation in a Drinking Water Reservoir

**DOI:** 10.3390/ijerph18062871

**Published:** 2021-03-11

**Authors:** Ji Yoon Kim, Usman Atique, Md Mamun, Kwang-Guk An

**Affiliations:** Department of Bioscience and Biotechnology, Chungnam National University, Daejeon 34134, Korea; jiyoonn20@naver.com (J.Y.K.); physioatique@gmail.com (U.A.); mamun1006001@gmail.com (M.M.)

**Keywords:** long-term water quality, nutrients, high flow, algal blooms, drinking water, sewage pollution, Daecheong Reservoir

## Abstract

Long-term variations in reservoir water chemistry could provide essential data in making sustainable water quality management decisions. Here, we analyzed the spatiotemporal variabilities of nutrients, sestonic chlorophyll-a (CHL-a), nutrient enrichment, dominant algal species, and overall chemical water health of the third-largest drinking water reservoir in South Korea during 2000–2020. Our results distinctly explained the strong influence of monsoon rainfall on spatial and annual water chemistry variations. We observed a consistent increase in the chemical oxygen demand alluding to organic matter pollutants, while a steady declining trend in the sestonic CHL-a. The long-term total phosphorus (TP) level showed a steady reduction from the riverine zone to the lacustrine area. However, a higher total coliform bacteria (TCB) was observed at the water intake tower sites. TP displayed a strong link to algal CHL-a and ambient nitrogen phosphorus ratios, suggesting a robust phosphorus-limitation state. The severe phosphorus-limitation was also corroborated by the findings of trophic state index deviation. The high and low flow dynamics exhibited the strong influence of intensive rainfall carrying many nutrients and sediments and flushing out the sestonic CHL-a. Successive eutrophic conditions prevailed along with dominating blue-green algae species (Microcystis and Anabaena). We observed a strong positive correlation (r = 0.62) between water temperature and CHL-a and between total suspended solids and TP (r = 0.65). The multi-metric water pollution index characterized the overall water quality as ‘good’ at all the study sites. In conclusion, the long-term spatiotemporal variabilities of the ecological functions based on the nutrient-CHL-a empirical models are regulated mainly by the intensive monsoon precipitation. The drinking water could become hazardous under the recurrent eutrophication events and chemical degradations due to uncontrolled and untreated inflow of sewage and wastewater treatment plant effluents. Therefore, we strongly advocate stringent criteria to mitigate phosphorus and organic pollutant influx for sustainable management of Daecheong Reservoir.

## 1. Introduction

Freshwater reservoirs are multifaceted ecological systems that are intermediate in a spectrum of rivers and natural lakes. Large freshwater reserves are indispensable for viable urban populations, environmental health, sustainable economic growth, and greater strategic importance as they provide drinking and irrigation water, recreation, catch fisheries, transportation, hydroelectric power generation, and flood control [1,2,3]. However, under the increasing anthropic stressors, reservoirs at the global scale are facing water quality degradation under the combined influence of climate change [4], urbanization [5], and agriculture [6], land use [7], precipitation, and river discharge [8], topography, and surface geology [9].

The human-made reservoirs differ from naturally existing lakes in several fundamental ways, such as less water residence time (WRT), frequent water level fluctuations, extended physical shape, and regular human interventions [10,11]. There are approximately 18,000 artificial reservoirs in South Korea. Several are located near large metropolitans and serve as sources of drinking water supply and help mitigate floods during intensive monsoon rainfall events. However, the large reservoirs are under severe water quality degradation and nutrient enrichment, which jeopardize human and ecosystem services [12].

Reservoir water quality is based on the dynamically fluctuating multiple water chemistry factors that usually help us either by approximation or deviation/degradation from the standards set for human and ecological purposes [13]. Such variations of significant water quality factors—such as total phosphorus (TP) and total nitrogen (TN), their ambient rations (TN:TP), chlorophyll-a (CHL-a), and total suspended solids (TSS) level—are vital if within the optimum ranges, conversely, could pose severe ecological problems upon deviance and become detrimental to aquatic life [14,15]. Based on such deviations, the decision-makers infer the water resource’s future use and fate and decide about the suitability and future measures to control the degradations [16,17].

An elevated nutrient level (N, P) as a result of nutrient loading, for instance, is mostly considered as a significantly important issue that could pose a hazard to the water resource ability in serving the desired ecological functioning [18]. The variations in nutrient cycling impact the reservoir water quality in numerous ways, such as increased chances of enrichment, intensive organic matter degradation triggering abrupt oxygen depletion, reduced water clarity due to surge in phytoplankton biomass, and sudden inflow or washing out phenomena of nutrients [19,20]. In each case, reservoir production and water transparency are significantly influenced by the fluctuating nutrient regime. Prolific nutrient enrichment stimulates rapid transformations to and from the mesotrophic, eutrophic, and hypertrophic conditions [2,19,21]. Furthermore, higher sedimentation rate and amassing allochthonous solids can also subsidize the eutrophication process and cause ecological degradations to the downstream connected lentic and lotic water systems [22,23,24].

Recurrent eutrophication events persist as a dire threat to freshwater reservoirs worldwide [25]. Shifts in the planktonic community composition and structure are used as a classic response to such nutrient enhancements [26,27]. Furthermore, surfacing algal species (*Microcystis* spp.) as blooms could also disrupt critical food-web processes, be potentially toxic, and are linked to eutrophication [19,28]. Therefore, investigating the consequences of various water chemistry pressures essentially includes trophic state assessment, physicochemical changes, and unprecedented damage to freshwater biodiversity [29,30,31]. Furthermore, deteriorating water quality could alter the typical structure and functioning of the aquatic ecosystems by directly or indirectly influencing the reservoir’s response to changing inclusive environmental conditions [32,33].

South Korean lentic ecosystems are strongly influenced by the intensive summer monsoon rainfall as well as help mitigate the flood intensity [34,35]. Increased inconsistency in rainfall patterns (drought and flood conditions) distinctly impacts the volume and frequency of inflowing and flushing out nutrients from the reservoirs and lakes [8]. Higher rainfall mediated inflows profusely deliver excessive solids, organic matter, and pollutants [36,37]. Therefore, to better understand the historic and imminent water quality fluctuations, monsoon rainfall impacts on the long-term reservoir water quality are inevitable. Furthermore, the increasing exploitation and variety of anthropic activities in the reservoir basin area, long-term monitoring of water quality variables could yield crucial insights into the temporal and spatial water quality fluctuations and nutrient enrichment status. These are the potential reasons we have undertaken this study.

Here we assessed the long-term water quality responses and trends over the past 20 years in a drinking water facility (Daecheong Reservoir) in South Korea during 2000–2020. We evaluated predominant water quality trends, spatial changes in selected water quality factors at the study sites, and water intake tower locations. Furthermore, we investigated the changing patterns of nutrients regime (N, P, and their ambient ratios) and the influence of monsoon rainfall intensity (drought and flood years). We also investigated the nutrient enrichment status and dominant trophic condition in the riverine (Rz), transitional (Tz), and lacustrine zone (Lz) through variations in trophic state index (TSI) and trophic state index deviation (TSID). The predominant links between algal chlorophyll-a (CHL-a) and nutrients and suspended solids as well as estimated the critically essential limiting nutrient during the long-term analysis. In the end, we appraised the prevalent nutrient pollution status at the reservoir sites and intake towers with a modified multi-metric water pollution index (WPI).

## 2. Materials and Methods

### 2.1. Study Area

**Figure 1:**
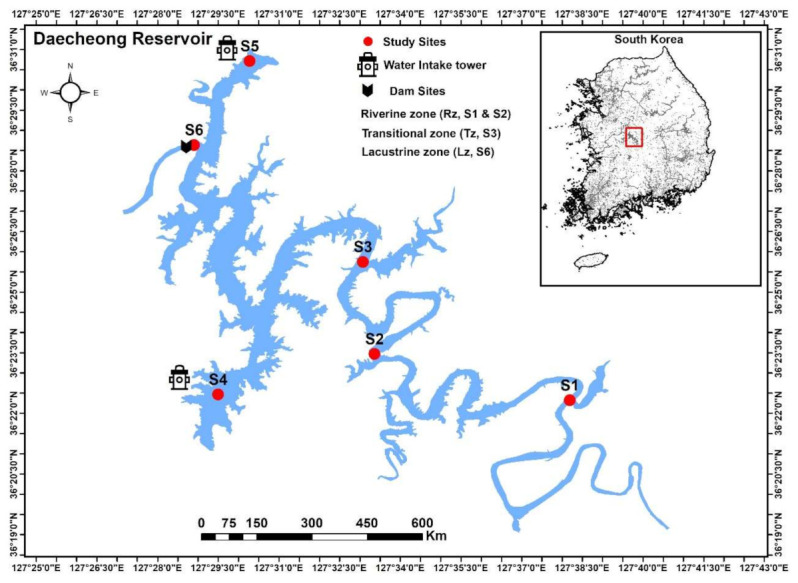
Study area map showing the sampling sites of the Daecheong Reservoir. S1 and S2: riverine zone (Rz); S3: transitional zone (Tz); S6: Lacustrine zone (Lz); S4 (IT1) and S5 (IT2): intake tower of drinking water.

Daecheong Reservoir (DR) is constructed on the third-largest river basin of South Korea (Geum River), is roughly 130 km southward from the Seoul Metropolitan (36°24′ N; 127°30′ E). The Geum River was impounded in December 1980 to finish constructing this third-largest drinking water and flood control structure. It is a multipurpose water resource that supplies 922,000 m^3^ of drinking water per day to a population of more than two million in its surrounding cities—including Daejeon, Cheongju, Gunsan, Iksan, and other adjacent communities. Apart from human consumption, it is a vital source of irrigation, hydroelectric power generation, flood mitigation, and recreation. It is morphologically complex, with a combined utilization of concrete gravity and embankment dam style construction, relatively stretched, narrow and dendritic in shape with a maximum 1 km width (Figure 1).

During maximum water supply, it has a volume of 1490 × 10^6^ m^3^, with a total surface area of 72.8 km^2^ and an extreme depth of 55 m. The geographic and other physiognomic features are shown in Table 1. The DR has the characteristic warm monomictic mixing, with a whole vertical mixing during winter, while steady stratification during other seasons. Typically, it undergoes large water level fluctuations up to 15 m during the summer monsoon intensive rainfall.

### 2.2. Study Sites and Longitudinal Zonation and Land Use Patterns

We divided the DR study sites into longitudinal zones as: S_1_ and S_2_ (riverine zone (Rz): 36.3743, 127.639, and 36.362, 127.592), S_3_ (transitional zone (Tz): 36.435, 127.553), S_6_ (lacustrine zone: dam site; 36.511, 127.503). The S_4_, and S_5_ are home to the water intake towers (IT_1_: 36.373, 127.553 and IT_2_: 36.475, 127.484), as shown in Figure 1. The Rz represents the headwater reservoir zone is the upper reach and is characterized by narrow width, relatively higher flow rates, and river-like morphology). The Lz, however, indicates the reservoir region representing the down lake area adjacent to the dam section (characterized by lower flow), and the Tz represents the intermediary features between those of Rz and Tz (relatively wider and intermediate water flow). The DR’s major feeding tributaries include Seowha, Bouchoung, Soung, Youngdong, Whebuk, and Janggae streams [12,35]. The site-based land use pattern could be described as S_1_ (40% rural area, 50% crop farming, 10% livestock), S_2_ (90% urban, 10% crop farming and openly subjected to the wastewater disposal plant (WWTP), S_3_ (45% crop farming, forest), S_4_, S_5_, and S_6_ (78% forest area and 22% rural area).

### 2.3. Water Quality Sampling and Rainfall Data

We studied 17 water quality factors at the chosen study sites during 2000–2020. The water samples were collected in standard sampling bottles from the surface to 50 cm depth in epilimnetic zone. The sampling bottles were covered and stored in the icebox immediately to prevent sunlight exposure. The rainfall data (2000–2020) were obtained from the Korean Meteorological Administration, while the monthly reservoir inflow and outflow records were procured from the Korean Water Resource Corporation. The WRT was defined from the water volume divided by inflow rate. The electrical conductivity (EC), dissolved oxygen (DO), water temperature (WT), hydrogen ion concentration (pH), total suspended solids (TSS) were determined at the spot using a portable multi-parameter analyzer (YSI Sonde Model 6600). The water clarity measured as the Secchi depth (20 cm metal disk; SD) was also measured on the spot. Total phosphorus (TP) and allied chemical species (TDP, PO_4_-P) were determined by the ascorbic acid method after persulfate oxidation, which was standardized by the Ministry of Environment, Korea [38]. Total nitrogen (TN) and similar parameters (NO_3_-N, NH_4_-N, and TDN) were estimated using the UV spectrophotometric method after the potassium persulfate digestion [39,40]. Biological oxygen demand (BOD) and chemical oxygen demand (COD) were determined by the Eatson and Franson [40] method, which the Ministry of Environment also standardized in South Korea. Chlorophyll-a (CHL-a) was measured using a spectrophotometer after extraction in acetone, based on the standardized Korean Ministry of Environment method [38]. The total coliform bacteria (TCB) were estimated according to the method of APHA [41]. To safeguard the precision in lab analysis, we measured nutrients (TP, TN) and CHL-a in duplicate, while BOD and COD were performed in duplicates [38,41].

### 2.4. Assessment of Algal Blooms and Species

The harmful blue-green algae were estimated by taking the 500 mL of the reservoir water sample and fixed with Lugol’s solution before transferring to the laboratory for species classification and identification. All specimens were identified according to the key characteristics mentioned by Chung [42]. For quantitative analysis, we used 1 mL of the water sample into the Sedgewick Rafter counting chamber to make observations of the fixed sample at 200–400 magnification using an optical microscope (Carl Zeiss, Oberkochen, Germany) based on the method approved by the National Institute of Environmental Research (NIER) [43].

### 2.5. Trophic State Index and Light Attenuation

The trophic state index (TSI) was evaluated by the natural logarithmic transformation (Ln) of CHL-a (µgL^−1^), SD (m), and TP (µgL^−1^) according to the following relations [44].

TSI (CHL-a, µgL^−1^) = 10 × [6 − (2.04 − 0.68 ln(CHL-a))/ln2] (1)

TSI (TP, µgL^−1^) = 10 × [6 − ln(48/TP)/ln2] (2)

TSI (SD, m) = 10 × [6 − ln(SD)/ln2](3)

The conventional criteria used to determine the prevalent eutrophication status based on the trophic state of TP, Chl-a, and SD. The average TSI range given for the oligotrophic is 30–40, mesotrophic 40–50, eutrophic 50–70, and hypereutrophic > 70 [45]. We inferred the predominant relations between the TSI of CHL-a, TP, and SD by plotting the deviations of the TSI, based on two-dimensional approaches of TSI (CHL-a) − TSI (SD) and TSI (CHL-a) − TSI (TP). This two-dimensional graphical approach is frequently used to illustrate the degree of eutrophication and the limiting nutrient status in reservoirs [46]. The water column light penetration is determined by various factors other than algal communities. Therefore, the calculation of the non-algal light attenuation coefficient (K_na_) estimates the water column light penetration. The K_na_ is calculated by the equation given by Walker [47].

K_na_ = 1/SD − 0.025 × CHL-a (4)

### 2.6. Water Pollution Index

We characterized the DR chemical health by applying the modified multi-metric index—i.e., water pollution index (WPI). It was altered from the nutrient pollution index (NPI) by Dodds et al. [48] for the USA and by Lee and An [49] in South Korea, and later on, modified by Atique and An [2]. It comprises seven metrics, covering major water chemistry factors that could be accounted for the ambient water pollution grade. It can be used for seasonal, spatial, and inter-annual water quality evaluations in both lentic and lotic ecosystems. The individual metrics (M_1–7_) and their units are M_1_: total nitrogen (mg/L); M_2_: total phosphorus (µg/L); M_3_: ambient ratios of TN:TP; M_4_: biological oxygen demand (mg/L); M_5_: total suspended solids (mg/L); M_6_: electrical conductivity (µS/cm); and M_7_: CHL-a (µg/L). The scoring benchmark authorized to each metric is established for ranges after evaluating the observed distribution of involved water quality factors. The given criteria for each metric (M) were either 5, 3, or 1, based on the observed values. Each site’s concluding chemical health status was calculated based on each metric’s obtained score and summing up all the scores. The final scores alluded to the conclusive grouping into excellent (31–35), good (25–29), fair (19–23), poor (13–17), and very poor (07–11).

### 2.7. Data Analysis

We run the Kolmogorov–Smirnov test to evaluate the data normality. The Rosner outlier test and Mann–Kendall Trend (MKT) test was performed by ProUCL (v 5.1) software [50]. Before the empirical regression study, the TP, TN, CHL-a, TSS, and SD were log-transformed to boost linear distribution. Sigma Plot (v.14.5) was used for regression plots. Pearson’s correlation analysis on the water chemistry and hydrological factors was performed in R studio. We determined the seasonal variability of blue-green algae during 2015–2020. The monsoon rainfall regime decided the seasonal variabilities as premonsoon (January–June), monsoon (July–August), postmonsoon (September–December). We also used the principal component analysis (PCA) to identify the multifaceted relationship between the water quality variables and hydrological factors. PCA helped reduce the data dimensions and divulged the data variance. PCA was conducted in SPSS (v. 24). The spatial analysis was performed with the help of Arc GIS (v. 10.4).

## 3. Results and Discussion

### 3.1. Inter-Annual Variations in Salient Water Chemistry Factors

**Figure 2:**
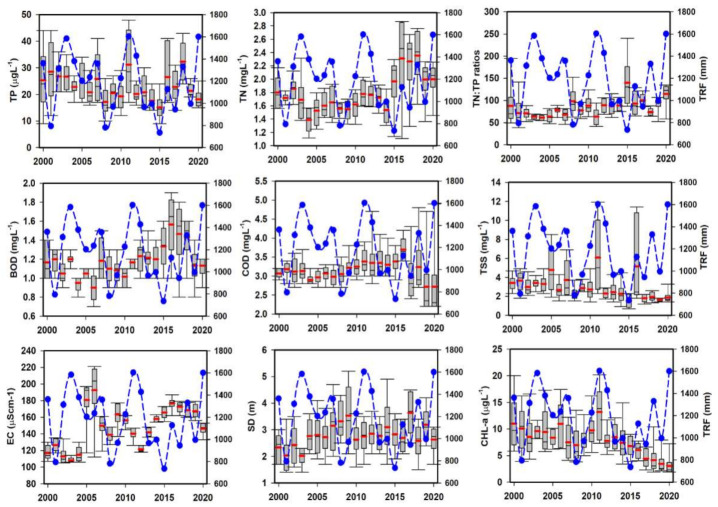
Inter-annual variation of salient water quality parameters. TP: total phosphorus, TN: total nitrogen, BOD: biological oxygen demand, COD: chemical oxygen demand, TSS: total suspended solids, EC: Electrical conductivity, SD: Secchi depth, CHL-a: chlorophyll-a. The blue curve line indicated the total rainfall (TRF) while the red lines indicate means.

The inter-annual variation in organic pollutants (COD, BOD), suspended solids (TSS), nutrients (TN, TP), and algal productivity (CHL-a) illustrated conspicuous heterogeneities due to the severe rainfall events during monsoon (Figure 2). The TN annual loads increased with the intensity of the precipitation. However, the TP level exhibited a remarkable increase during the intense monsoon during 2011, the flood year in the reservoir watershed and the Korean peninsula. The rainfall influenced the ambient nutrient ratios (TN:TP) inversely as the TP level increased with the rain. The mean TP level displayed a good approximation with the rainfall patterns during the study. Generally, the BOD levels fluctuated diversely with the rainfall; however, COD demonstrated a steady increase during 2000–2018, with a sharp decline in 2019–2020. It suggested the gradual tendency of decrease in oxygen-demanding chemical pollutants in the reservoir that could indicate the change in anthropogenic activities, especially industrial production.

A consistent rise of chemical contaminants in lentic water bodies must pose grave threats to the water resource’s intended usages, which in this case is mostly for drinking [2,6]. The results indicated a growing influx of oxygen-demanding chemicals generated by the various anthropic and geochemical processes in the reservoir basin [8]. The annual TSS showed a mixed response to high and low inflows mediated by intensive precipitation. Although CHL-a disclosed a similar development as TP, it is essential to mention an overall decline in the annual CHL-a levels during the study period, mostly post 2015 drought year it is showed a sharp decline. A valid reason for this decline could be the successive increase in annual rainfall that could have washed out most of the algal CHL-a [12]. TSS disclosed varied loads to the yearly fluctuating precipitation. Such remarkably varying loadings of TSS, TN, TP, EC, and algal CHL-a provided the decisive influence of precipitation intensity both as a single event and the total rainfall (TRF) during the study. The flood years mostly indicated an increase in the nutrient-contributing parameters, while drought years displayed a decline. It could be inferred that the lower rainfall intensity could have helped in the reduction of nutrient-rich inflows that could be potentially linked to the lower production of the sestonic CHL-a [8,51]. However, according to Brasil et al. [20], the reduction in reservoir water level (reduced inflow of water currents) favors the production of cyanobacterial blooms that could be linked to higher algal productivity.

### 3.2. Spatial Fluctuations in Water Quality

**Figure 3:**
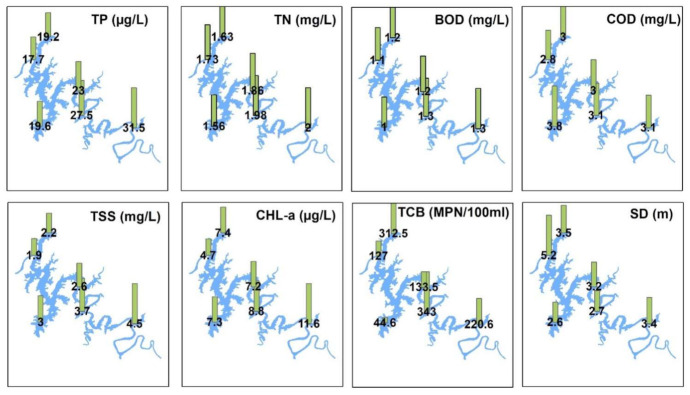
Spatial variations of water quality parameters at the reservoir zones and water intake towers. TP: total phosphorus, TN: total nitrogen, BOD: biological oxygen demand, COD: chemical oxygen demand, TSS: total suspended solids, CHL-a: chlorophyll-a, TCB: total coliform bacteria, SD: Secchi depth. For site identification, please refer to Figure 1.

The spatial examination of the longitudinal zones and water intake towers of the DR based on annual averages of the vital water chemistry factors including TN, TP, BOD, TSS, CHL-a, and TCB demonstrated conspicuous variations (Figure 3). The TP level was higher in the first site of the Rz (31.52 µg/L) that gradually dropped to 17.71 µg/L at the Lz, which is also the dam site. The TN, however, did not fluctuate much at the different zones and water intake towers. BOD was the lowest at the IT_1,_ while the highest average value was observed at the Rz. The COD level was the highest at the IT_1_ (3.84 mg/L) while the lowest at the IT_2_ (2.8 mg/L). It indicated higher risks of organic pollutants in the drinking water supply that could be highly hazardous to the consumers, especially for long term usage. However, the TSS and SD displayed an inverse relationship with the highest TSS at the Rz, which could be attributed to the turbulent flows exceptionally high rainfall mediated. The algal CHL-a average showed more significant variance with the highest level at S_1_ (11.55 µg/L), while the lowest annual average was observed at the dam site—i.e., S_6_ and represents the Lz (4.7 µg/L).

The total coliform bacteria (TCB) showed interesting and higher fluctuations along the longitudinal reservoir gradient. The highest (312.47 MPN mL/100) load of TCB was observed at the IT_2_ while the lowest (44.61 MPN mL/100) at the IT_1_. Although the average levels are not up to the hazardous limits, these findings indicate the fluctuating levels that could potentially erupt some bacterial diseases, especially in children, if proper and regular monitoring is not carried. TCB is usually used as a reliable indicator of sewage pollution in freshwater reservoirs, especially for those used for drinking and swimming purposes [2]. There is a higher possibility of contracting waterborne bacterial diseases with higher bacterial abundance in drinking water facilities [52]. Therefore, TCB’s regular monitoring is of broader concern due to higher waterborne diseases risks [53].

### 3.3. Flood and Drought Impacts of Nutrient Regime, Organic Pollutants, CHL-a, and TCB

**Figure 4:**
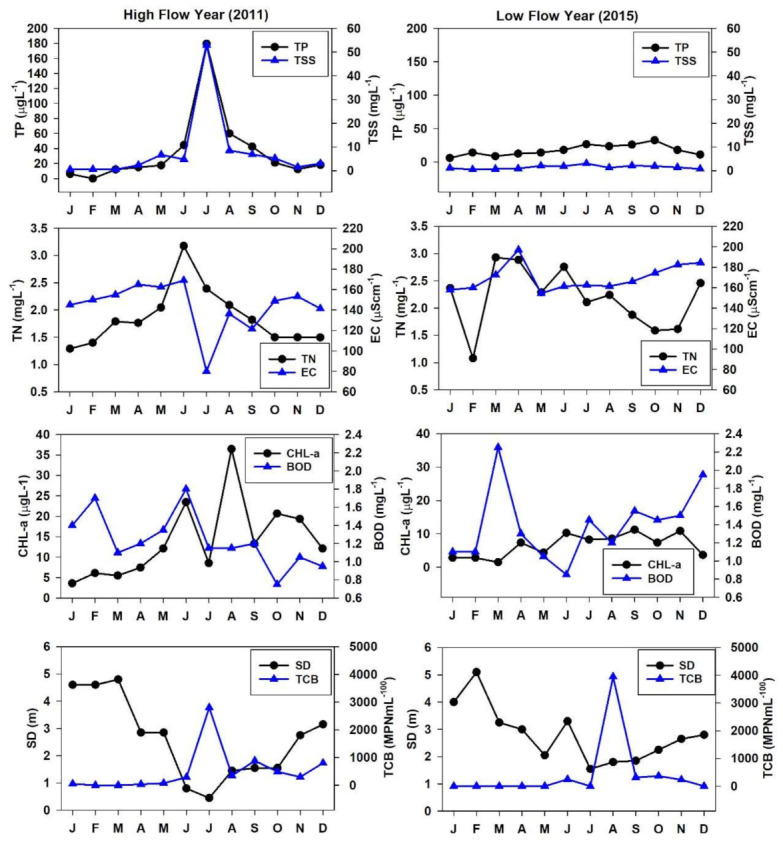
Monthly variations of selected water quality parameters during the high (2011) and low (2015) flow years. TP: total phosphorus, TSS: total suspended solids, TN: total nitrogen, EC: electrical conductivity, CHL-a: chlorophyll-a, BOD: biological oxygen demand, SD: Secchi depth, TCB: total coliform bacteria.

The seasonal and annual rainfall patterns primarily influence Korean freshwater resources. Therefore, we compared vital water quality factors during the high flow (2011) and low flow (2015) years. The TP and TSS approximated the seasonal rainfall intensity during the high flow year while displayed almost a steady level during the low flow year. This alluded to the highly manipulating impact of the intensive rainfall events and how it delivers higher levels of nutrients and solid contents to the reservoir ecosystem (Figure 4). The TN showed an increase while the EC demonstrated dilutions during the high flow year while showed comparatively higher loads during the low flow. In the case of algal CHL-a, it approximated the BOD levels, which showed dilutions during monsoon, while CHL-a illustrated high productivity during and postmonsoon months (July–September). However, during low flow year, CHL-a showed a higher peak spring season on the same magnitude during the monsoon while BOD did not show substantial influence. However, the TCB showed an abrupt increase during the high rainfall events irrespective of the high and low flow years. Fluctuating flows run by the rainfall intensity lead to flood and drought conditions that may vary in each case.

The studies based on flood and drought dynamics have become even more significant due to the abruptly changing climatic conditions [54], and increasing anthropogenic impacts and South Korea is at a higher risk of climate change. Therefore, it is incredibly essential to study the potential effects of high and flow years on vital water chemistry factors. The water quality fluctuations become even critical high flows [55,56]. Frequent and severe high and low flow conditions may negatively influence the WRT, stable movement of nutrients, aquatic biodiversity, and damage to costly installations [8]. Therefore, high and flow investigations are more appropriate in DR owing to their frequent and broader use.

### 3.4. Predominant Relations between Water Chemistry and Hydrology

**Figure 5:**
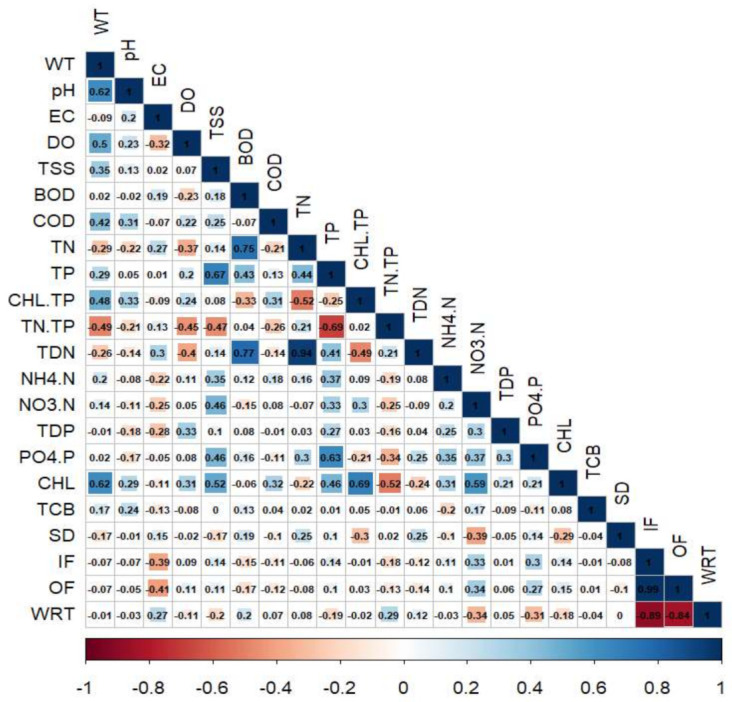
Pearson correlation analysis on water quality parameters during 2000–2020. WT: water temperature, pH: hydrogen ion concentration, EC: electrical conductivity, DO: dissolved oxygen, TSS: total suspended solids, BOD: biological oxygen demand, COD: chemical oxygen demand, TN: total nitrogen, TP: total phosphorus, TDN: total dissolved nitrogen, NH_4_-N: ammonium-nitrogen, NO_3_-N: nitrate-nitrogen, TDP: total dissolved phosphate, PO_4_-P: phosphate, CHL: chlorophyll, TCB: total coliform bacteria, SD: Secchi depth, IF: inflow, OF: outflow, WRT: water residence time. The graphs’ color scale is explained as red color range showing negative while blue color range positive correlation.

The Pearson’s correlation assessment on all water quality parameters, inflow, outflow and WRT yielded mostly week positive to moderately strong positive links between water quality factors and flow regime in DR during 2000–2020 (Figure 5). We determined the strength of correlation as weak when r ≥ 0.30–≤ 0.49, moderately strong when r ≥ 0.50–≤ 0.69, strong as r ≥ 0.7. However, we considered no significant correlation when r < 30. The WT indicated a moderately strong (r = 0.62) relationship with pH and CHL-a, while EC illustrated a weak negative (r = −0.41) association with the reservoir outflow. The suspended solids (TSS) indicated moderately strong association with TP (r = 0.67) and algal CHL-a (r = 0.52). It showed that the TP is largely contributed by the agricultural activities in the watershed absorbed in the inflow sediments from agricultural lands during the intensive rainfall events [7,36]. The BOD levels directly increased with the increasing loads of TN (r = 0.75) and TDN (r = 0.77), while COD mostly showed very week to no relations with most of the critical parameters. This showed a declining trend in the chemical pollutants in the DR, which is a good sign for its water quality for drinking purposes. Among the interactions of nutrients (TN, TP) and algal CHL-a, TP showed a weak positive (r = 0.42) association with CHL-a, while TN showed negative to minimal correlation. The hydrological parameters (inflow, outflow, and WRT) mostly did not tangibly influence critical water quality factors.

### 3.5. Long-Term Trend Analysis

We used the Mann–Kendall trend (MKT) analysis to detect the prevalent trends among the vital factors of reservoir water quality as well as to illustrate the sustainable usability of the DR. For this, we implemented the trend investigation on the 20 years of the dataset of each parameter as MKT is a non-parametric test. The MKT rendered meaningful awareness about the extant tendencies in crucial water quality regulatory determinants in the DR (Table 2). The parameters we discussed include the water pH, WT, EC, DO, TSS, BOD, COD, TP, TN, ambient nutrient ratios, CHL-a, etc. There is an increasing trend in the EC, BOD, TN, TDN, and bacterial indicators (total coliform bacteria; TCB). This shows that the reservoir water quality is jeopardized for its sustainable resources as a drinking water facility. It also alludes to the consistently increasing anthropic actions in the reservoir watershed as well as the increasing pressure of urban populations. Possible reasons for growing trends in TN and TDN could support the notion that nitrogen is present is in a surplus amount in the ecosystem while the allied chemical species (NO^3^-N and NH_4_-N), especially nitrates, could be used in phytoplankton growth. The other factors could be fluctuating temperature and inflow. The other reasons could include the changes in these chemical species owing to various chemical reactions taking place.

In contrast, we detected no trend in the pH, COD, TP, and allied chemical species (PO_4_-P, TDP). This is a good sign for drinking water facility that indicated a decline of the inflowing chemical pollutants, which could have increased the DO levels, helping sustain the aquatic life [14]. Furthermore, it marked a potential decline in the TP yielding fertilizers in the reservoir watershed. There is a decreasing tendency in the influx of TSS, WT, ammonia, and nitrates, along with the algal CHL-a level. This alluded to the links between suspended solids, water clarity, and primary productivity.

Furthermore, the decline of suspended solids enhanced the water transparency measured as SD. This inference is further reinforced by the opposite tendencies in the TSS and EC. These findings based on the MKT strongly advocate for the skillful management of various pressures and human activities, particularly from the urban areas located in the DR watershed [24]. It is essential to mention that the prevalent trend detection is based on historic data and does not necessarily imply stringent future trends, especially under rapidly changing climatic conditions [57].

### 3.6. Empirical Relationship between Nutrients, Algal CHL-a, Ambient Ratios, and Clarity

**Figure 6:**
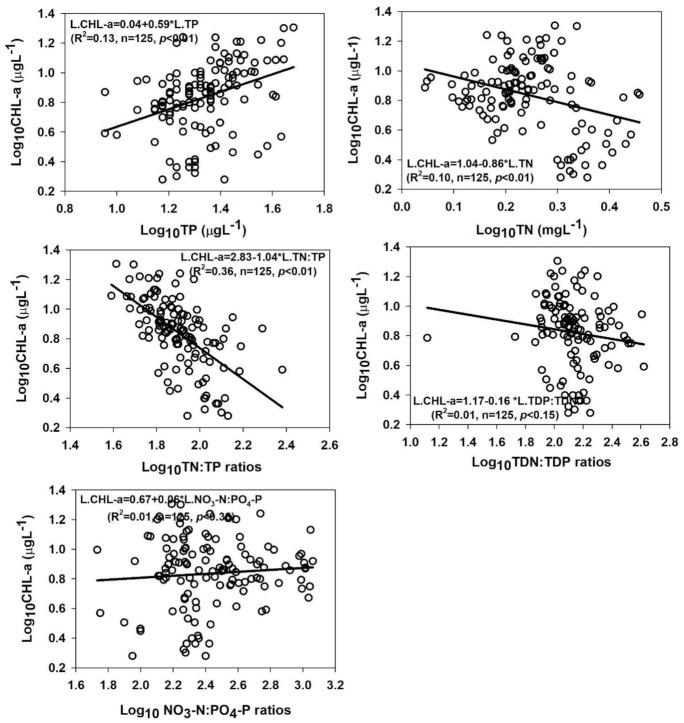
Empirical relationships between chlorophyll-a, nutrients, and their ambient ratios. CHL-a: chlorophyll-a; TP: total phosphorus; TN: total nitrogen; TDN: total dissolved nitrogen; TDP: total dissolved phosphorus; NO_3_-N: nitrate-nitrogen; and PO_4_-P: phosphate.

**Figure 7:**
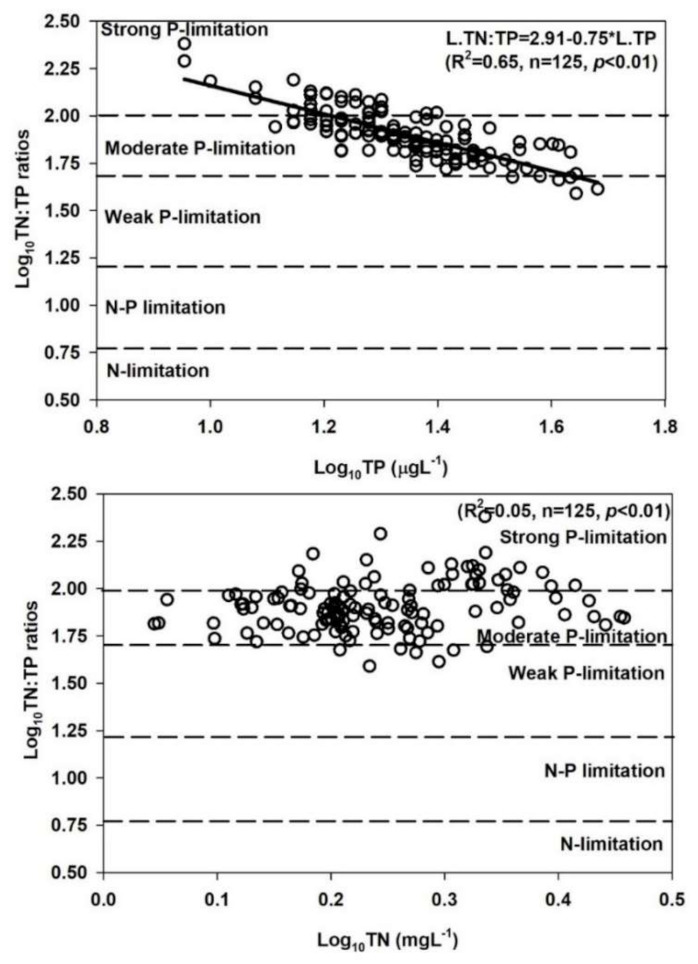
Determination of limiting nutrient based on empirical modeling on the TN:TP ratios, TP, and TN. TP: total phosphorus; TN: total nitrogen.

We determined the most limiting nutrient for the primary productivity in the DR, and the results demonstrated TP could be used as a better predictor for algal CHL-a production (Figure 6). However, the empirical modelling on the log-transformed parameters suggested that the ambient N:P ratios could help understand the predominant limiting nutrient (R^2^ = 0.36, *p* < 0.01) in the present study. In order to see if TP actually is the limiting nutrient, the N:P ratios displayed strong relationship (R^2^ = 0.65, *p* < 0.01) with TP, while almost no relationship with the TN (R^2^ = 0.05, *p* < 0.01). However, both the relation displayed severe TP limitation in the reservoir (Figure 7).

**Figure 8:**
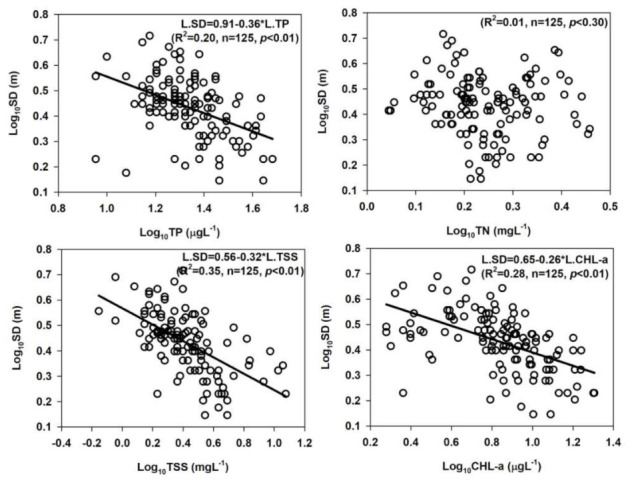
Empirical links between water transparency (SD), nutrients. TP: total phosphorus; TN: total nitrogen; suspended solids (TSS), and chlorophyll-a (CHL-a) in Daecheong Reservoir during 2000–2020.

**Figure 9:**
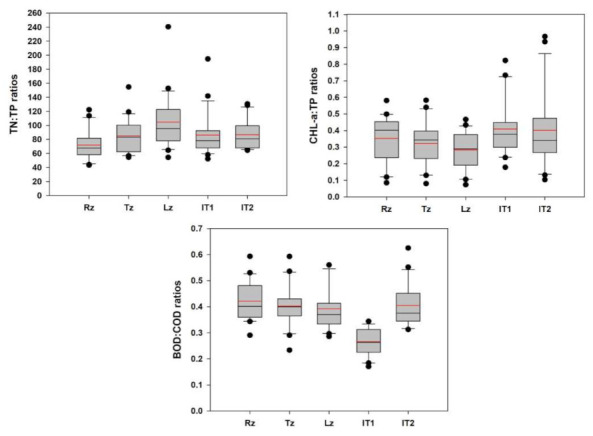
Dynamics of nutrients, algal chlorophyll-a, and organic pollutant ratios in longitudinal zones of Daecheong Reservoir. TN: total nitrogen; TP: total phosphorus; CHL-a: chlorophyll-a; BOD: biological oxygen demand; COD: chemical oxygen demand; Rz: riverine zone; Tz: transitional zone; Lz: Lacustrine zone; IT1 and IT2: intake tower of drinking water 1 and 2; red lines in the box indicate mean values.

We plotted the SD with nutrients, CHL-a and TSS and the results indicated that the water clarity was primarily determined by TP (R^2^ = 0.20) and CHL-a (R^2^ = 0.28) levels after the TSS (R^2^ = 0.35) in the reservoir (Figure 8). The ambient ratios of TN:TP, CHL-a:TP, and BOD:COD displayed spatial heterogeneities in the longitudinal reservoir zones and water intake towers (Figure 9). These results approximate the spatial dynamics of nutrients, algal CHL-a and organic pollution indicators in reservoir zones and water intake tower sites. This is important to mention the due to the almost no relation with TN, the chances of collinearity are minor, and the system is strongly P-limited [12,35,58].

### 3.7. Trophic Status Evaluation and Blue-Green Algae Blooms

**Figure 10:**
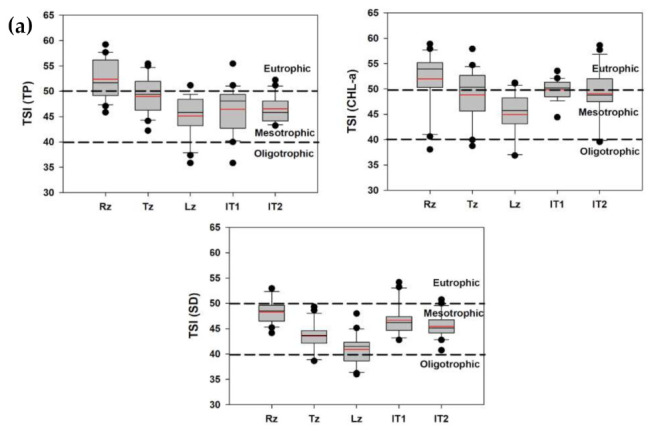

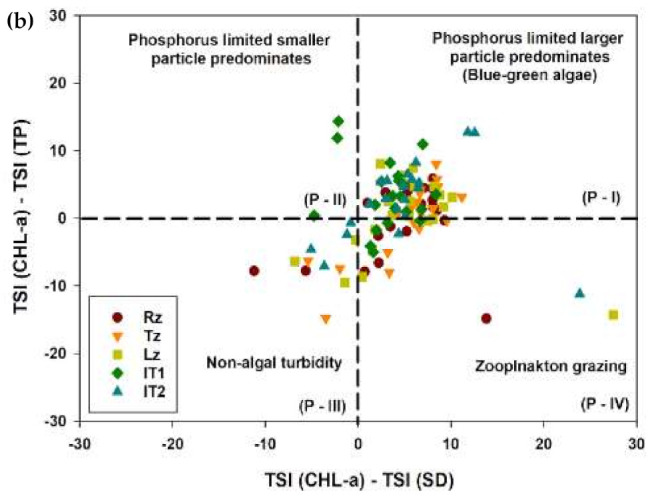
Trophic state index (**a**) and its deviation (**b**) based on TP, CHL-a and SD of Daecheong Reservoir. TP: total phosphorus; CHL-a: Chlorophyll-a; SD: Secchi depth; Rz: riverine zone; Tz: transitional zone; Lz: Lacustrine zone; IT_1_ and IT_2_: intake tower of drinking water 1 and 2; red lines in the box indicate mean values.

During recent decades, moderate to severe nutrient enrichments have emerged as a significant problem for water quality managers worldwide [59]. The leading reason for high eutrophication is increasing TP and TN loads in lakes and reservoirs due to the overwhelming agricultural and industrial actions and the untreated effluent discharge from WWTPs. To check the nutrient enrichment status, we employed the trophic state indices (TSI). The TSI (TP), CHL-a, and SD displayed a spatial decline in the nutrient enrichments from the Rz to Lz in the Daecheong Reservoir (Figure 10a). The Rz mostly showed the eutrophic status while the other two zones were at a mesotrophic level. The water intake towers, however, remained in the mesotrophic state as well.

Most of the reservoirs and lakes face severe water quality degradations due to the nutrient influx, turbidity, and harmful algal blooms (HABs) that could influence their designated utilization. Therefore, a potential trend of the impending eutrophication and HABs could render valuable evidence for sustainable water quality management [8]. Consequently, we studied the trophic state index deviation (TSID) in the reservoir zones and water intake towers. The two-dimensional graphical illustration based on Carlson’s [44] method severe P-limitation and higher chances of blue-green algae (Figure 10b).

**Figure 11:**
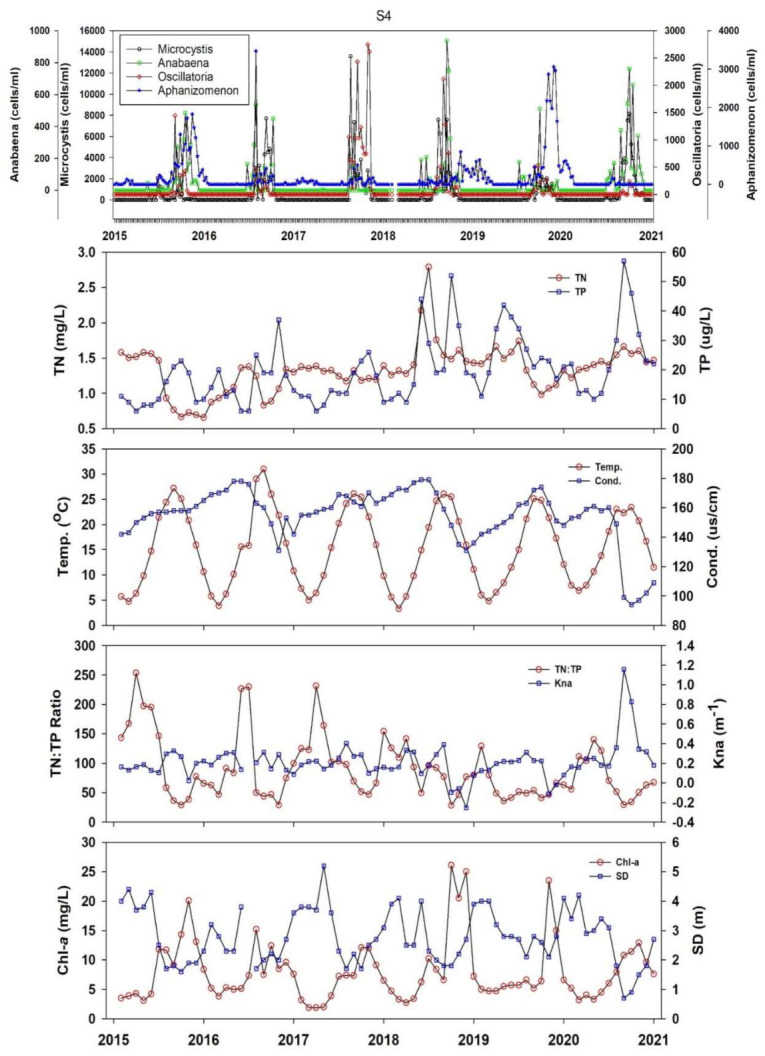
Relationship between the algal blooms of four blue-green algae species, nutrients, ambient ratios, water temperature, and algal CHL-a during 2015–2020.

**Figure A1:**
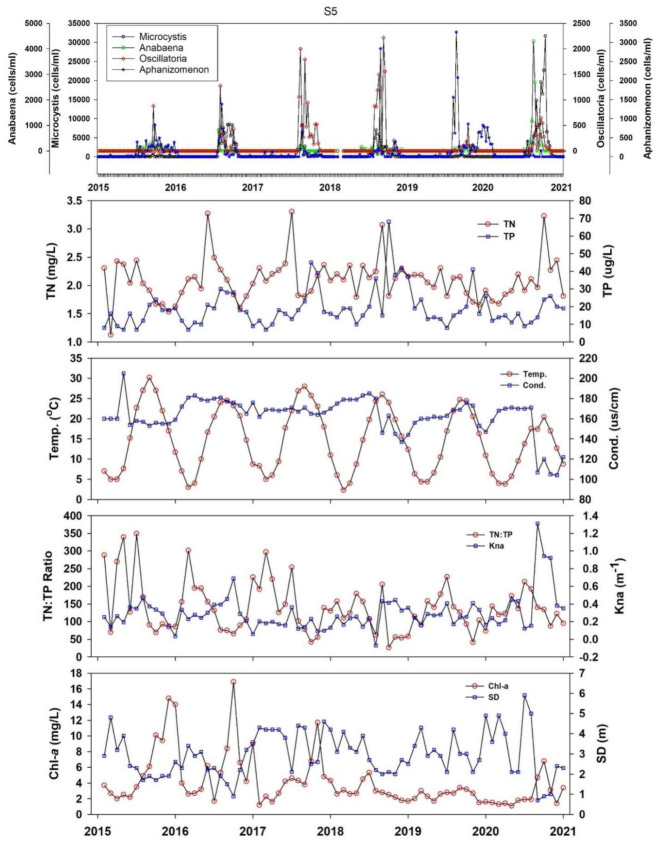
Comparison between the blue-green algae species, nutrients, water temperature and non-algal light attention coefficient (Kna) during 2015–2020 at the IT_2,_ i.e., Site 5.

There are slight zooplankton grazing chances (18%); however, non-algal turbidity in approximately all the reservoir zones could be anticipated for a short time. The larger particles and blue-green algae’s apparent tendency alluded to a higher likelihood of recurrent eutrophication events. To check the presence of blue-green algae, we examined the presence of four blue-green algae species viz. *Anabaena*, *Microcystis*, *Oscillatoria*, and *Aphanizomenon* during 2015–2020 and checked if any other nutrient, CHL-a, or temperature showed approximations. The results are illustrated in Figure 11. It denoted that the blue-green algal species increased with the increasing TP loads, water temperature, non-algal light attenuation (K_na_) factor and CHL-a at Site 4, i.e., is the water intake tower 1. For intake tower 2, the relationship between the same parameters and algal species is presented in Appendix A Figure A1. The trophic state index deviation illustrated severe P-limitation and higher chances of algal blooms directly peaking with the higher TP loads, CHL-a productivity, and increasing WT. These findings demonstrated that the DR is under the severe threat of recurrent HABs that could seriously jeopardize human health. Therefore, it is imperative to mitigate the inflowing TP levels into the DR by regulating the P-yielding fertilizers and the industrial activities that could potentially contribute to higher phosphorus levels.

### 3.8. Principal Component Analysis

**Figure 12:**
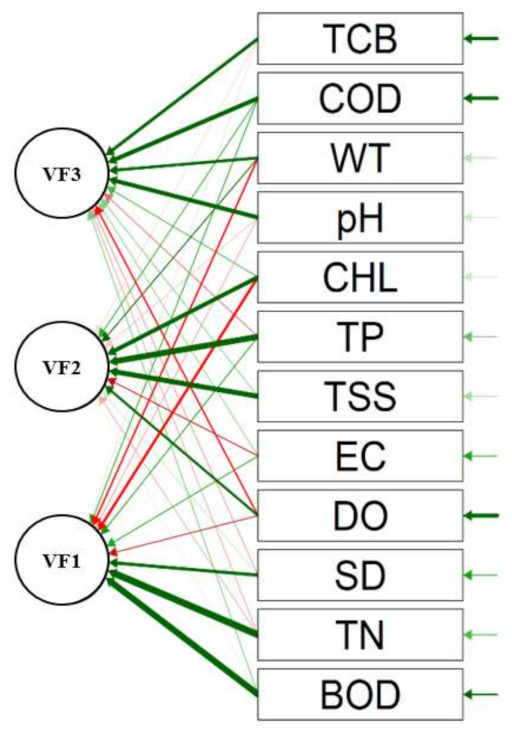
Path diagram of water quality parameters for principal component analysis.

To reduce the dimensionality and check the variance in the water quality factors and dominant factors impacting the reservoir water quality, we performed the principal component analysis combined with factor analysis (PCA/FA) and the results are shown in Table 3. The path diagram indicating the predominant links between the water quality factors is presented in Figure 12. We performed the Bartlett’s test and checked the KMO (Kaiser–Meyer–Olkin) value to examine the factor analysis’s data suitability (PCA/FA). Our results indicated that data was suitable as the KMO value was 0.49. Simultaneously, the Bartlett’s test showed significance (*p* < 0.000), and we found some redolent links between the dominant water chemistry parameters and potentially causing agents/factors in the reservoir watershed. The PCA with varimax rotation recognized three varifactors (VFs), explaining 65.56% of the cumulative variance (CV).

The VF1 accounted for 26.04% of the CV, and disclosed a robust positive loading (>0.70) value [2,60] of BOD and TN with moderate negative loading (−0.55) of algal CHL-a as well as 0.60 loadings of SD. Such high loadings alluded to the dominance of organic matter pollutants and their negative impact on algal CHL-a productivity during the study period. It further denoted that the organic matter (BOD) positively increased with the increasing TN loadings. It also signified the increasing domestic sewage and mounting agricultural activities in the reservoir water basin. However, the VF2 (21.20% variance) characterized the higher loadings of TSS, TP, and algal CHL-a showing the prevalent positive associations between the TP inflows with the higher influx of solids with the likelihood of carrying the phosphorus from the agricultural watershed. It further signified the TP as the limiting nutrient for sustainable algal productivity in the reservoir. The third VF (18.32% variance) displayed the increasing impact of industrial effluents and WWTPs as the pH and COD loadings were observed as strong positive loadings. The moderately strong loading of TCB (0.61) also strengthened the notion that the reservoir is increasingly receiving the untreated WWTP effluents, and three is a tendency to increase industrial activities. These findings are richly supported by previous studies [6,7,8,31,60].

### 3.9. Overall Water Pollution Status

We determined the overall chemical water health status at the reservoir zones (Rz, Tz, Lz) and water intake towers (IT_1_, IT_2_) in DR during 2000–2020, and the results are displayed in Table 4. The modified WPI was applied to estimate the reservoir zonal chemical health status that indicated an overall ‘good’ chemical health at all sites, except a little lower score at the Rz. To demonstrate the WPI, we considered a total of seven water quality metrics signifying the most prominent factors. The WPI is used to entitle the predominant chemical health status of freshwater resources that can be used flexibly, such as seasonal, spatial, and annual trends. The underlying concept of WPI is that vital water chemistry factors could be used as indicators of chemical degradation by evaluating the water quality changes showing either deviation or approximation to the standards of critical elements [31,61].

The required water chemistry parameters characterize the nutrients (TN, TP, TN:TP ratios), organic pollutants (BOD), solid and ionic contents (TSS and EC), and primary productivity measured in terms of CHL-a. Based on annual averages of the nutrient regime, the TN represented the mesotrophic (1.5–3.0 mg/L) nutrient enrichment while the TP showed an oligotrophic (<30 µg/L) state at all the study sites, including water intake towers. The organic pollutants (BOD) and ionic (EC) levels showed no significant variations. In contrast, the suspended solids (TSS) showed a moderate level (4–10 mg/L) at the Rz as compared to the other zones and intake sites. The highest level of algal CHL-a at the Rz showed slight degradation compared to other sites. The outcomes indicated all zones and water intake towers in good chemical health (29 scores) with an Rz score of 25. Previous research richly supports these results as well [2,8,61,62].

## 4. Conclusions

In conclusion, we performed a long-term water quality assessment in the third-largest drinking water resource in South Korea during 2000–2020. We studied the interannual and spatial variabilities in salient water quality factors along the longitudinal zones (Rz, Tz, Lz) and the water intake tower (IT_1_, IT_2_). We also determined the dynamics of high and low flow based on the flood and drought conditions mediated by the summer monsoon. The spatial, seasonal, and annual comparisons between algal CHL-a and nutrients displayed heterogenic responses. The reservoir is under severe P-limitation corroborated by the empirical modelling between nutrients, ambient N:P ratios, and algal CHL-a responses.

Furthermore, the N:P ratios also provide practical reasons to be accounted for a P-limitation scenario. The MKT analysis showed an increasing tendency in the TCB to result in higher sewage-mediated pollution and waterborne diseases. Similar is the case with the growing trends in BOD, while algal CHL-a displayed a decreasing trend during the past 20 years. The monsoon rainfall intensity of the most important driving factor in the influx of solids, TP, and flushing out of algal CHL-a. The SD is mostly characterized by the TP and TSS loads along with the primary productivity. The high and low flow years displayed conspicuous variations among the nutrients and CHL-a levels that could be further studied.

The DR water intake tower sites mostly exhibited the mesotrophic state. Simultaneously, longitudinal zonation displayed a continuous decline in the nutrient enrichment from the Rz to Lz that alluded to higher WRT’s influence on settling down solids and nutrients. The TSID evaluation indicted the dominance of larger particles and blue-green algae with an indication of severe P-limitation. We also identified the dominant harmful algal species and the observations revealed that the Anabaena, *Microcystis*, *Oscillatoria*, and *Aphanizomenon* are likely to bloom during the postmonsoon period. These blooms mostly followed TP’s higher inflows, growing primary productivity levels, and increasing water temperature. The multi-metric WPI designated all the reservoir zones and water intake towers in a ‘good’ chemical health. Overall, the Daecheong Reservoir has shown stable and good water quality status during our long-term analysis. However, high and low flows are decisive and prevalent spatial heterogeneities in the organic and sewage pollution indicators alluded to higher risks of waterborne diseases in the water consumers. Furthermore, there is an increasing tendency of HABs in the future due to the dominating blue-green species. The monsoon regime’s powerful influence will be critical to the spatiotemporal water quality changes, eutrophication, and high and low flow dynamics.

## Figures and Tables

**Table 1:** Physical dimensions and hydrological characteristics of Daecheong Reservoir.

Hydrological and Dimensional Attributes	Characteristics
Construction period	1975–1980
Dam type	Concrete gravity and embankment dam
Height of dam	72 m
Length of dam	495 m
Mean depth	21.2 m
Maximum depth	69 m
Average water residence time	180 days
Surface area	6.8 × 10^7^ m^2^
Surface water volume	14.3 × 10^8^ m^3^
Average annual rainfall	1400 mm
Flood water level	80 m
Full reservoir level	76.5
Total capacity	14.9 × 10^8^ m^3^
Active capacity	79 × 10^7^ m^3^
Hydroelectric power generation	90 MW

**Table 2:** Long-term trend analysis of water quality parameters based on Mann–Kendall trend (MKT) test in Daecheong Reservoir during 2000–2020.

Parameters	S Value	*p* Value	Slope	Intercept	Trend	Trend Sign
pH	7	0.42	−0.0007	7.77	No trend	0
WT	−67	0.02	−0.1	15.49	Decreasing trend	-
EC	80	0.00	2.11	125.27	Increasing Trend	+
DO	−95	0.00	−0.05	9.98	Decreasing trend	-
TSS	−103	0.00	−0.07	3.86	Decreasing trend	-
BOD	70	0.00	0.01	1.02	Increasing trend	+
COD	29	0.19	0.002	3.11	No trend	0
TP	−54	0.05	−0.2	25.34	No trend	0
TN	78	0.01	0.02	1.49	Increasing trend	+
TN:TP	96	0.01	1.82	64.46	Increasing trend	+
TDP	1	0.5	0.0001	0.011	No trend	0
PO_4_-P	−51	0.06	−0.0001	0.006	No trend	0
NO_3_-N	−98	0.00	−0.01	1.31	Decreasing trend	-
NH_4_-N	−90	0.00	−0.0018	0.06	Decreasing trend	-
TDN	130	0.00	0.03	1.19	Increasing trend	+
CHL	−140	0.00	−0.31	11.35	Decreasing trend	-
CHL:TP	−66	0.02	−0.01	0.46	Decreasing trend	-
SD	82	0.00	0.16	1.58	Increasing trend	+
TCB	71	0.01	12.83	55.63	Increasing trend	+

pH: hydrogen ion concentration; WT: water temperature EC: electrical conductivity; DO: dissolved oxygen; TSS: total suspended solids; BOD: biological oxygen demand; COD: chemical oxygen demand; TP: total phosphorus; TN: total nitrogen; TDP: total dissolved phosphate; PO_4_-P: phosphate; NO_3_-N: nitrate-nitrogen; NH_4_-N: ammonium- nitrogen; TDN: total dissolved nitrogen; CHL: chlorophyll; SD: Secchi depth; TCB: total coliform bacteria.

**Table 3:** Principal component analysis of water quality parameters.

Variables	Components		
	VF1	VF2	VF3
pH	−0.15	−0.09	**0.70**
WT	−0.46	0.41	*0.58*
DO	−0.36	*0.54*	−0.45
EC	0.36	−0.39	0.18
TSS	−0.01	**0.77**	0.20
BOD	**0.89**	−0.03	0.22
COD	0.37	0.34	**0.71**
TN	**0.93**	−0.13	−0.14
TP	0.39	**0.84**	−0.23
CHL	−*0.55*	**0.71**	0.30
TCB	−0.05	−0.06	*0.61*
SD	0.60	0.08	−0.16
Eigenvalues	3.12	2.54	2.19
% of Variance	26.04	21.20	18.32
Cumulative %	26.04	47.24	65.56

Varimax rotation method, bold and italic represents strong (>0.70) and moderate positive loadings (0.5–0.70); pH: hydrogen ion concentration; WT: water temperature; DO: dissolved oxygen; EC: electrical conductivity; TSS: total suspended solids; BOD: biological oxygen demand; COD: chemical oxygen demand; TN: total nitrogen; TP: total phosphorus; CHL-a: Chlorophyll-a; TCB: total coliform bacteria; SD: Secchi depth).

**Table 4:** Chemical health assessment of Daecheong Reservoir using multi-metric water pollution index (WPI) during 2000–2020.

Category	Model Metrics	Scoring Criteria			Reservoir Zone			Intake Tower	
		5	3	1	Rz	Tz	Lz	IT1	IT2
Nutrient Regime	M_1_: TN (mg/L)	<1.5	1.5–3.0	>3	1.98 (3)	1.85 (3)	1.72 (3)	1.55 (3)	1.63 (3)
	M_2_: TP(µg/L)	<30	30–100	>100	29.5 (5)	23.04 (5)	17.71 (5)	19.61 (5)	19.19 (5)
	M_3_:TN:TP ratio	>50	20–50	<20	72.13 (5)	85.17 (5)	104.81 (5)	86.16 (5)	86.84 (5)
Organic matter	M_4_: BOD (mg/L)	<1	1–2.5	>2.5	1.30 (3)	1.18 (3)	1.08 (3)	0.99 (3)	1.20 (3)
Ionic content and solids	M_5_: TSS (mg/L)	<4	4–10	>10	4.11 (3)	2.64 (5)	1.90 (5)	2.96 (5)	2.23 (5)
	M_6_: EC (µS/cm)	<180	180–300	>300	155.66 (5)	151.80 (5)	147.61 (5)	133.66 (5)	146.80 (5)
Primary production indicator	M_7_: CHL (µg/L)	<3	3 to 10	>10	10.18 (1)	7.24 (3)	4.72 (3)	7.28 (3)	7.4 (3)
Final Scores					25	29	29	29	29
Water Quality Criteria					Good	Good	Good	Good	Good

Rz: riverine zone; Tz: transitional zone; Lz: Lacustrine zone; IT1 and IT2: intake tower of drinking water 1 and 2.

## Data Availability

The data may be available upon request to the corresponding author, subject to the funding agency’s approval.

## References

[1] Mahdiyan O., Filazzola A., Molot L.A., Gray D., Sharma S. (2021). Drivers of water quality changes within the Laurentian Great Lakes region over the past 40 years. Limnol. Oceanogr..

[2] Atique U., An K.-G. (2019). Reservoir Water Quality Assessment Based on Chemical Parameters and the Chlorophyll Dynamics in Relation to Nutrient Regime. Pol. J. Environ. Stud..

[3] Znachor P., Nedoma J., Hejzlar J., Seďa J., Kopáček J., Boukal D., Mrkvička T. (2018). Multiple long-term trends and trend reversals dominate environmental conditions in a man-made freshwater reservoir. Sci. Total Environ..

[4] Michalak A.M. (2016). Study role of climate change in extreme threats to water quality. Nature.

[5] Lee S.W., Hwang S.J., Lee S.B., Hwang H.S., Sung H.C. (2009). Landscape ecological approach to the relationships of land use patterns in watersheds to water quality characteristics. Landsc. Urban Plan..

[6] Mamun M., Kwon S., Kim J.E., An K.G. (2020). Evaluation of algal chlorophyll and nutrient relations and the N:P ratios along with trophic status and light regime in 60 Korea reservoirs. Sci. Total Environ..

[7] Atique U., An K.-G. (2020). Landscape heterogeneity impacts water chemistry, nutrient regime, organic matter and chlorophyll dynamics in agricultural reservoirs. Ecol. Indic..

[8] Hara J., Atique U., An K.G. (2020). Multiyear links between water chemistry, algal chlorophyll, drought-flood regime, and nutrient enrichment in a morphologically complex reservoir. Int. J. Environ. Res. Public Health.

[9] Kopacek J., Stuchlık E., Straskrabova V., Psenakova P. (2000). Factors governing nutrient status of mountain lakes in the Tatra Mountains. Freshw. Biol..

[10] Hayes N.M., Deemer B.R., Corman J.R., Razavi N.R., Strock K.E. (2017). Key differences between lakes and reservoirs modify climate signals: A case for a new conceptual model. Limnol. Oceanogr..

[11] Li Z., Ma J., Guo J., Paerl H.W., Brookes J.D., Xiao Y., Lunhui L. (2018). Water quality trends in the Three Gorges Reservoir region before and after impoundment (1992–2016). Ecohydrol. Hydrobiol..

[12] Mamun M., Kim J.Y., An K.-G. (2020). Trophic Responses of the Asian Reservoir to Long-Term Seasonal and Interannual Dynamic Monsoon. Water.

[13] Hutchins M.G., Abesser C., Prudhomme C., Elliott J.A., Bloomfield J.P., Mansour M.M., Hii O.E. (2018). Combined impacts of future land-use and climate stressors on water resources and quality in groundwater and surface waterbodies of the upper Thames river basin, UK. Sci. Total Environ..

[14] Atique U., An K.-G. (2018). Stream health evaluation using a combined approach of multi-metric chemical pollution and biological integrity models. Water.

[15] Haque M.A., Jewel M.A.S., Atique U., Paul A.K., Iqbal S. (2020). Seasonal and spatial variation of flagellate communities in a tropical river. Limnologica.

[16] Logan B., Taffs K. (2013). Relationship between diatoms and water quality (TN, TP) in sub-tropical east Australian estuaries. J. Paleolimnol..

[17] Trochine C., Guerrieri M., Liboriussen L., Willems P., Lauridsen T.L., Søndergaard M., Jeppesen E. (2017). Factors controlling the stable isotope composition and C: N ratio of seston and periphyton in shallow lake mesocosms with contrasting nutrient loadings and temperatures. Freshw. Biol..

[18] da Rocha Junior C.A.N., da Costa M.R.A., Menezes R.F., Attayde J.L., Becker V. (2018). Water volume reduction increases eutrophication risk in tropical semi-arid reservoirs. Acta Limnol. Bras..

[19] Jeppesen E., Brucet S., Naselli-Flores L., Papastergiadou E., Stefanidis K., Nõges T., Nõges P., Attayde J.L., Zohary T., Coppens J. (2015). Ecological impacts of global warming and water abstraction on lakes and reservoirs due to changes in water level and related changes in salinity. Hydrobiologia.

[20] Brasil J., Attayde J.L., Vasconcelos F.R., Dantas D.D.F., Huszar V.L.M. (2016). Drought induced water-level reduction favors cyanobacteria blooms in tropical shallow lakes. Hydrobiologia.

[21] Schindler D.W. (2012). The dilemma of controlling cultural eutrophication of lakes. Proc. R. Soc. B Biol. Sci..

[22] Markad A.T., Landge A.T., Nayak B.B., Inamdar A.B., Mishra A.K. (2019). Trophic state modeling for shallow freshwater reservoir: A new approach. Environ. Monit. Assess..

[23] Hughes R.M., Dunham S., Maas-Hebner K.G., Yeakley J.A., Schreck C., Harte M., Schaeffer J. (2014). A review of urban water body challenges and approaches: (1) Rehabilitation and remediation. Fisheries.

[24] Atique U., Kwon S., An K.-G. (2020). Linking weir imprints with riverine water chemistry, microhabitat alterations, fish assemblages, chlorophyll-nutrient dynamics, and ecological health assessments. Ecol. Indic..

[25] Le Moal M., Gascuel-Odoux C., Ménesguen A., Souchon Y., Étrillard C., Levain A., Pinay G. (2019). Eutrophication: A new wine in an old bottle?. Sci. Total Environ..

[26] Haberman J., Haldna M. (2014). Indices of zooplankton community as valuable tools in assessing the trophic state and water quality of eutrophic lakes: Long term study of Lake Võrtsjärv. J. Limnol..

[27] Havens K.E., Ansari A.A., Gill S.S. (2014). Lake Eutrophication and Plankton Food Webs. Eutrophication: Causes, Consequences and Control.

[28] Paerl H.W., Otten T.G. (2013). Harmful cyanobacterial blooms: Causes, consequences, and controls. Microb. Ecol..

[29] Jackson M.C., Loewen C.J., Vinebrooke R.D., Chimimba C.T. (2016). Net effects of multiple stressors in freshwater ecosystems: A meta-analysis. Glob. Chang. Biol..

[30] Kim J.Y., Atique U., An K.-G. (2021). Relative Abundance and Invasion Dynamics of Alien Fish Species Linked to Chemical Conditions, Ecosystem Health, Native Fish Assesmblage, and Stream Order. Water.

[31] Kim J.-J., Atique U., An K.-G. (2019). Long-Term Ecological Health Assessment of a Restored Urban Stream Based on Chemical Water Quality, Physical Habitat Conditions and Biological Integrity. Water.

[32] Smith V.H., Schindler D.W. (2009). Eutrophication science: Where do we go from here?. Trends Ecol. Evol..

[33] Atique U., Byungjin L., Johee Y., An K.-G. (2019). Biological Health Assessments of Lotic Waters by Biotic Integrity Indices and their Relations to Water Chemistry. Water.

[34] An K.G., Park S.S. (2002). Indirect influence of the summer monsoon on chlorophyll-total phosphorus models in reservoirs: A case study. Ecol. Model..

[35] An K.-G., Park S.S., Ahn K.Y., Urchin C.G. (2003). Dynamics of nitrogen, phosphorus, algal biomass, and suspended solids in an artificial lentic ecosystem and significant implications of regional hydrology on trophic status. J. Environ. Biol..

[36] An K.G., Kim D.S. (2003). Response of reservoir water quality to nutrient inputs from streams and in-lake fishfarms. Water. Air Soil Pollut..

[37] Atique U., Iqbal S., Khan N., Qazi B., Javeed A., Anjum K.M., Haider M.S., Khan T.A., Mahmood S., Sherzada S. (2020). Multivariate Assessment of Water Chemistry and Metals in a River Impacted by Tanning Industry. Fresenius Environ. Bull..

[38] MOE (2000). Standard Methods for the Examination of Water Quality Contamination.

[39] Crumpton W.G., Isenhart T.M., Mitchell P.D. (1992). Nitrate and organic N analyses with second-derivative spectroscopy. Limnol. Oceanogr..

[40] Eaton A., Franson M.A. (2005). Standard Methods for the Examination of Water and Wastewater.

[41] APHA (2005). Standard Methods for the Examination of Water and Wastewater.

[42] Chung J. (1993). Illustration of the Freshwater Algae of Korea.

[43] NIER (2013). Changes of Water Environment and Phytoplankton Community Structures in the Nakdong River.

[44] Carlson R.E. (1977). A trophic state index for lakes. Limnol. Oceanogr..

[45] Carlson R.E., Simpson J. (1996). A Coordinator’s Guide to Volunteer Lake Monitoring Methods.

[46] Carlson R.E., Havens K.E. (2005). Simple graphical methods for the interpretation of relationships between trophic state variables. Lake Reserv. Manag..

[47] Walker W.W. (1982). An empirical analysis of phosphorus, nitrogen, and turbidity effects on reservoir chlorophyll-A levels. Can. Water Resour. J..

[48] Dodds W.K., Jones J.R., Welch E.B. (1998). Suggested classification of stream trophic state: Distributions of temperate stream types by chlorophyll, total nitrogen and phosphorus. Water Res..

[49] Lee H.J., An K.-G. (2009). The development and application of multi-metric water quality assessment model for reservoir managements in Korea. Korean J. Limnol..

[50] Singh A., Maichle R. (2016). ProUCL V. 5.1.Statistical Software for Environmental Applications for Data Sets with and without Nondetect Observations.

[51] Deitch M.J., Dolman B. (2017). Restoring Summer Base Flow under a Decentralized Water Management Regime: Constraints, Opportunities, and Outcomes in Mediterranean-Climate California. Water.

[52] Sood A., Singh K.D., Pandey P., Sharma S. (2008). Assessment of bacterial indicators and physicochemical parameters to investigate pollution status of Gangetic river system of Uttarakhand (India). Ecol. Indic..

[53] Hoyer M.V., Donze J.L., Schulz E.J., Willis D.J., Canfield D.E. (2006). Total coliform and escherichia coli counts in 99 florida lakes with relations to some common limnological factors. Lake Reserv. Manag..

[54] Bhagwat T., Klein I., Huth J., Leinenkugel P. (2019). Volumetric Analysis of Reservoirs in Drought-Prone Areas Using Remote Sensing Products. Remote Sens..

[55] Han Z., Huang S., Huang Q., Leng G., Wang H., He L., Fang W., Li P. (2019). Assessing GRACE-based terrestrial water storage anomalies dynamics at multi-timescales and their correlations with teleconnection factors in Yunnan Province, China. J. Hydrol..

[56] Ren K., Huang S., Huang Q., Wang H., Leng G., Fang W., Li P. (2020). Assessing the reliability, resilience and vulnerability of water supply system under multiple uncertain sources. J. Clean. Prod..

[57] Yue S., Pilon P., Cavadias G. (2002). Power of the Mann ± Kendall and Spearman s rho tests for detecting monotonic trends in hydrological series. J. Hydrol..

[58] Jones J.R., Knowlton M.F. (2005). Chlorophyll response to nutrients and non-algal seston in missouri reservoirs and oxbow lakes. Lake Reserv. Manag..

[59] Palácio S.M., Espinoza-Quiñones F.R.E., de Pauli A.R., Queiroz C.B., Fabris S.C., Fagundes-Klen M.R., Veit M., Piana P.A. (2016). Assessment of anthropogenic impacts on the water quality of Marreco River, Brazil, based on principal component analysis and toxicological assays. Water Air Soil Pollut..

[60] Muangthong S., Shrestha S. (2015). Assessment of surface water quality using multivariate statistical techniques: Case study of the Nampong River and Songkhram River, Thailand. Environ. Monit. Assess..

[61] Kanownik W., Policht-Latawiec A. (2015). Changeability of oxygen and biogenic indices in waters flowing through areas under various anthropopressures. Pol. J. Environ. Stud..

[62] Taranu Z.E., Gregory-Eaves I., Steele R.J., Beaulieu M., Legendre P. (2017). Predicting microcystin concentrations in lakes and reservoirs at a continental scale: A new framework for modelling an important health risk factor. Glob. Ecol. Biogeogr..

